# Can Unicellular Organisms Sequester a Germline? The Yeast‐Germline Hypothesis

**DOI:** 10.1002/bies.70003

**Published:** 2025-05-02

**Authors:** Bhavya Sree Vadlamudi, Duur K. Aanen

**Affiliations:** ^1^ Plant Sciences Group, Laboratory of Genetics Wageningen University Wageningen the Netherlands; ^2^ Agrotechnology & Food Sciences Group, Laboratory of Systems and Synthetic Biology Wageningen University Wageningen the Netherlands

**Keywords:** evolution, germline, germline‐sequestration, immortal strand hypothesis, *Saccharomyces cerevisiae*, soma, Weismann's germplasm theory

## Abstract

Germline mutations can affect future generations, while somatic mutations cannot. This germline‐soma distinction does not seem to make sense for unicellular organisms. We challenge this view, arguing that baker's yeast (*Saccharomyces cerevisiae*) has a germline. Under aerobic conditions yeast cells use mainly fermentation of glucose to produce ethanol. Only when glucose is exhausted, cells switch to full respiration of the produced ethanol. We hypothesize that only a subset of the cells continue dividing and switch to respiration. A change from exponential to linear growth is consistent with asymmetrical cell division, where a senescing mother cell produces quiescent daughter cells. We thus propose that most cells produced during fermentation are “somatic,” that is, they rapidly lose reproductive capacity, while the cells continuing to divide constitute the germline, as they exclusively produce rejuvenated quiescent cells. We discuss biased DNA‐template strand inheritance by the mother cell as a potential adaptive explanation for germline sequestration to reduce the mutation rate.

## Graphical abstract

Asymmetrical inheritance of template‐DNA and copy‐DNA strands during mitosis can reduce the number of mutant cells. This requires that cells continuing to divide inherit the “immortal” template‐DNA, while their daughter cells become quiescent and inherit copy‐DNA strands. We propose this DNA‐level germline sequestration for yeast cells after the diauxic shift.

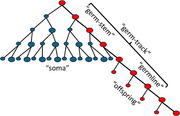

## 1 Introduction

**FIGURE 1:**
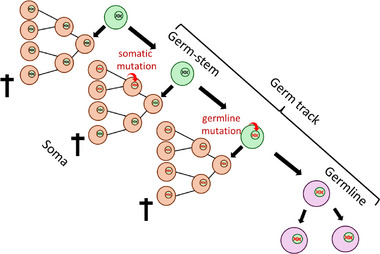
Illustration of the consequences of mutations occurring in the germline and soma. The germ track includes the germline and the germ‐stem cells. Germ‐stem cells (indicated in green) can produce both germ and somatic cells, while the germline (indicated in light purple) exclusively produces germ cells. Somatic cells are indicated in light orange. Mutations (curved red arrows) occurring in the germ stem and germline cells are inherited by the progeny (depicted as red DNA), whereas mutations occurring in somatic cells are not. + indicates that somatic cells die and do not leave any offspring.

**FIGURE 2:**
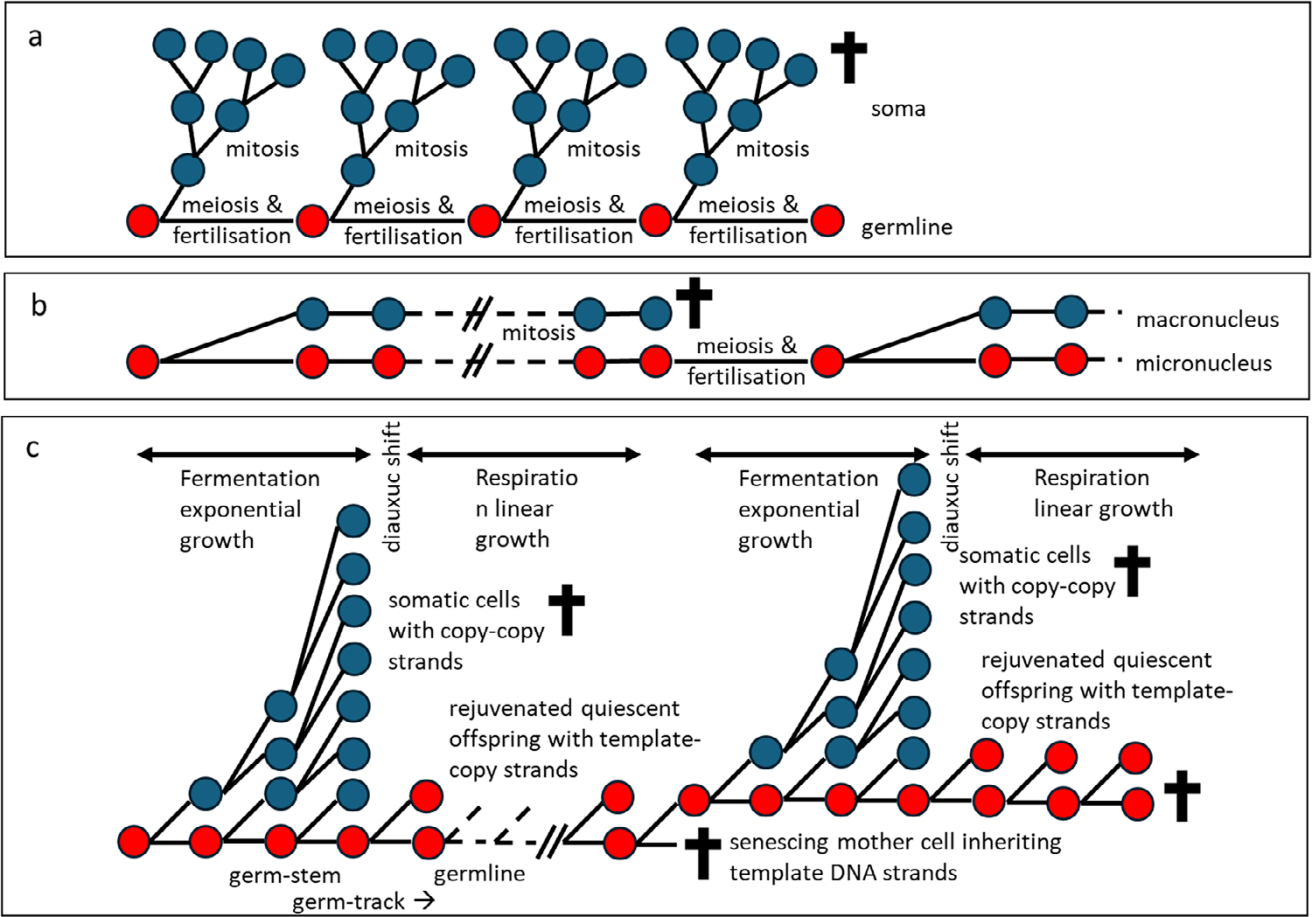
Illustration of the germline segregation in various organisms. (a) In animals, early germline sequestration with few germline‐cell divisions ensures that the number of mutations in germline cells is reduced. (b) Some ciliate protozoans reduce the number of mutations by having a transcriptionally silent so‐called micronucleus, next to a transcriptionally active macronucleus. After a number of mitotic divisions, the micronucleus undergoes meiosis, upon which one of the four haploid micronuclei undergoes a mitotic division and the other three deteriorate. Now one of the two remaining micronuclei fertilizes a different micronucleus of the same or of a different cell, and becomes fertilized by one of the two micronuclei of that cell. The then‐formed diploid micronucleus undergoes three mitotic divisions to form eight micronuclei. Then the cell undergoes two binary fissions to form four cells with two micronuclei. One of those micronuclei then becomes a new macronucleus that replaces the old macronucleus. (c) The theoretical model predicted to reduce the mutation rate in unicellular organisms worked out for budding yeast. After an exponential growth phase, some mother cells keep on dividing, producing daughter cells that stop dividing. If the dividing mother cells preferentially inherit template‐DNA strands, and the daughter cells copy strands, this results in a lower number of mutant cells than with random DNA strand inheritance. We hypothesize that the exponential and linear growth phases in budding yeast correspond to fermentative and respiratory growth, respectively, separated by the diauxic shift.

Weismann recognized the germ track (Keimbahn) as the cellular path by which germplasm is passed, unchanged, from parents to progeny [1]. We now know that the germplasm, ultimately, is the genetic information encoded in the genome. Further, David Haig divided the germ track into germ stem, cells with somatic and gametic descendants, and germline, cells exclusively committed to becoming gametes [2]. The distinction between germline and soma is evolutionarily significant because it distinguishes heritable mutations that directly affect the progeny from those that do not (Figure 1). The germline‐soma distinction forms the basis for evolutionary theories of aging [3]. Since most mutations are deleterious, natural selection is predicted to minimize the number of mutations in the germline [4, 5]. Some animals achieve this by early germline sequestration (Figure 2a). In plants, the germline is not sequestered early in development; instead, somatic cells in specific tissues go on to produce reproductive cells later in the plant's life cycle. However, plants reduce the number of cell divisions in the top meristems that form the flowers [6, 7].

Weismann proposed that unicellular organisms, similar to germline cells, are theoretically immortal. Interestingly, later studies have demonstrated that senescence does occur in unicellular organisms, even in those with morphologically symmetrical division such as *Escherichia coli* [8], and fission yeast (*Schizosaccharomyces pombe*) [9]. In those organisms, there appears to be a fundamental asymmetry between the cells resulting from a cell division, with one accumulating damaged cellular components, resulting in a decline of replication ability of that cell lineage, and the other cell being rejuvenated [8].

Although asymmetries during cell division seem to be universal, both daughter cells retain the ability to divide. Since the germ track is defined as a cellular path, it seems impossible to distinguish a germline from a soma for unicellular organisms [10]. Ciliates demonstrate that germline sequestration can also occur at the nucleus level, with a transcriptionally silent micronucleus that forms the germline and a transcriptionally active macronucleus that is a somatic cul‐de‐sac [11] (Figure 2b). For other unicellular organisms, it has earlier been proposed that segregating the template DNA strands in one cell lineage that continues to divide, and the copy strands in the daughter cell that stops dividing will reduce the mutation rate (Figure 2c) [12]. If correct, this determination based on DNA inheritance would be a more fundamental distinction between the germline and the soma. In this paper, we work out this hypothesis for budding yeast (*Saccharomyces cerevisiae*).

### 1.1 Biology of Yeast

Budding yeast is a unicellular eukaryote with a haploid chromosome number of 16 and can exist both in a haploid and a diploid state [13]. The diploid phase is the predominant state of yeast in nature and diploid cells can increase in numbers by mitotic division. Mitotic division occurs asymmetrically, by budding off a small “daughter” cell from a large “mother” cell. Under optimal conditions, yeast cells can divide every 100 min, but growth rates vary between haploid and diploid strains and between environments. The average replicative lifespan is about 26 cell divisions [14].

During clonal expansion, yeast cells initially use mostly fermentation of carbon sources like glucose, producing ethanol as a by‐product. In glucose‐rich media, growth is exponential with a fast doubling time. However, when glucose becomes depleted in the medium, cellular metabolism changes to respiration only, with catabolism of the produced ethanol. This is done via the tricarboxylic acid (TCA) cycle and oxidative phosphorylation in the mitochondria [15]. The metabolic re‐modeling from (anaerobic) fermentation to (aerobic) respiration is known as the diauxic shift [15]. After the respiratory phase, cells enter the post‐diauxic growth phase, characterized by a low specific growth rate [16, 17].

### 1.2 Yeast‐Germline Hypothesis

We hypothesize that yeast has a dedicated germline, which is differentiated from the soma. (For a glossary of terms, please see Box 1). The yeast‐germline hypothesis has two essential components: the first is about the growth phases of yeast cells and the second about DNA‐strand inheritance during the linear growth phase. According to our hypothesis, there is an exponential phase, during which mostly somatic cells are produced, and a linear phase, during which offspring cells are produced. As we will argue below, most cells produced during initial fermentative growth die and do not leave any offspring when growth conditions deteriorate, while a small fraction of the cells continue to divide after the switch to respiration, producing quiescent rejuvenated “offspring” cells. The first fraction, therefore, can be considered “somatic,” while the latter fraction constitutes a true germline, since those cells exclusively produce offspring and no somatic cells in the present generation (Figure 2c).

The second part of the yeast‐germline hypothesis is that germline cells preferentially retain template‐DNA strands after DNA replication and become the senescing mother cells, while their daughter cells receive the new copy strands and are rejuvenated [18, 19, 20]. Considering mutations that arise due to replication errors, template strands remain mutation‐free while copy strands can have replication errors. During the exponential phase, both cells resulting from a division produce on average, roughly equal numbers of descendants (see Supporting Information). As a result, irrespective of whether they inherit the template or copy strands, there is no apparent reduction in the resulting number of mutant cells. However, when growth is linear, it *does* matter which of the two daughter cells inherits the template strands. If the germline cells—defined as the dividing mother cells exclusively committed to producing offspring cells—inherit the template strands, the number of mutant cells will be reduced, since mutant copy strands will be only one cell division away from the template strands [18]. Paradoxically, therefore, the germline formed during the linear phase senesces but remains relatively mutation‐free. We first provide evidence for the growth part of the yeast‐germline hypothesis, and subsequently, for asymmetries in template and copy‐strand inheritance.

### 1.3 Linear Growth, Cell‐Level Senescence, and Quiescent Cell Formation During Respiration

**FIGURE 3:**
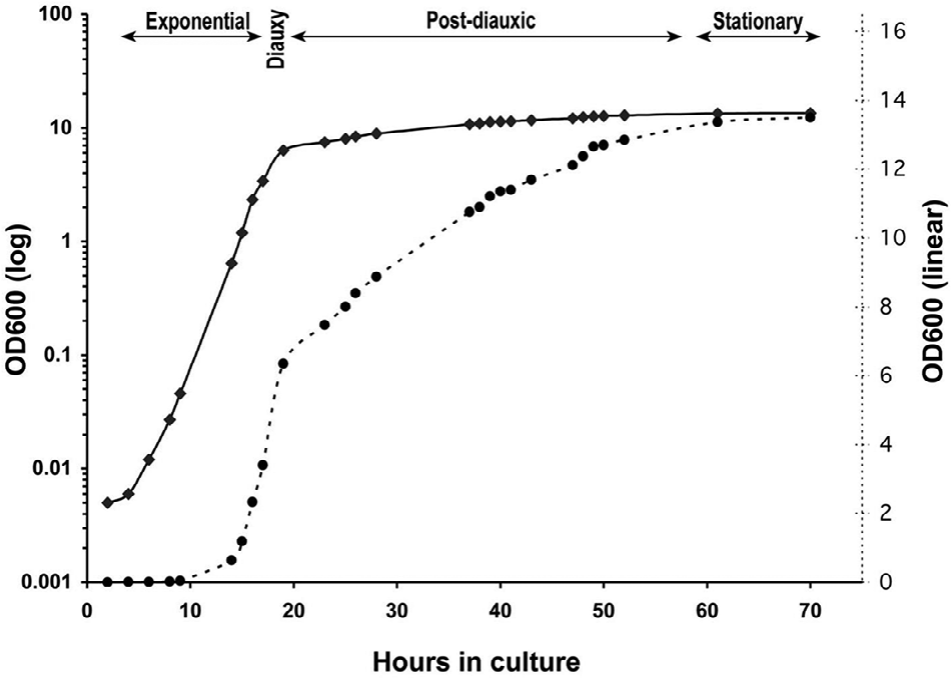
Growth curves of yeast cells grown in YPD medium for 70 h. Yeast cells grew exponentially for 20 h indicated by arrows. Following the diauxic shift, yeast cells switch to ethanol respiration and display a lower growth rate. The solid line is on a log scale, while the dotted line is on a linear scale. Adapted from Ref. [16].

A crucial component of the yeast‐germline hypothesis is that there is a linear growth phase. Close inspection of growth curves shows that post‐diauxic growth is linear, roughly leading to a doubling in population size, and is followed by the stationary phase [21, 22, 23] (Figure 3). Assuming a single founding cell of replicative age zero, and optimal culture conditions for exponential growth, the founding cell and its offspring cells can then divide 26 times, giving rise to 67.1 million cells [14]. The effect of cell‐level senescence on population growth is negligible, since at that point in time, the founding cell of the 67.1 million cells produced will die due to senescence (see Supporting Information). Please note that under natural ecological conditions, the potential for the exponential growth of yeast is constrained by limited resources and space [24]. It seems likely, therefore, that non‐exponential growth is the norm under most natural conditions.

For a doubling in population size, as approximately observed after the exponential growth, on average, each cell must divide once after the exponential phase. Notably, this process spans several days, which either means an unusually prolonged duration for a single cell division or irregular occasional divisions of the cells. An alternative, and we think more plausible, hypothesis is that a subset of the cell population continues to grow in a linear fashion. Linear growth occurs if cells produce daughter cells that do not undergo further divisions. Depending on the replicative age of the cells that enter linear growth, a certain fraction of the cells is required to achieve a doubling of the population size after the diauxic shift. For example, if cells that enter linear growth have replicative age zero, they can produce 26 daughter cells. This means that 1/26th of the population would need to enter linear growth to achieve a doubling in population size.

Several studies have shown that during the linear phase, a quiescent cell fraction is formed. These cells are rejuvenated and can start to divide if the medium is refreshed [25]. According to our hypothesis, the cells that continue dividing linearly after the diauxic shift thus exclusively produce germ cells and can be considered as a germline lineage (Figure 2c). Quiescence refers to a non‐proliferative state of cells characterized by low metabolic activity, also called the G_0_ phase [26]. Quiescent (Q) cells can be morphologically distinguished from non‐quiescent (NQ) cells by being smaller and rejuvenated, having higher resilience, and a higher stress tolerance. They exhibit high reproductive competence, that is, when they are allowed to grow in conditions that promote growth, they proliferate and form new colonies. They can also re‐enter the cell‐division cycle synchronously when supplied with fresh nutrients [27]. In contrast, the non‐quiescent fraction formed by cells of the first and later generations continues to divide. These cells have low reproductive ability and undergo senescence in the later generations [27]. Consistent with the yeast germline hypothesis, a Q cell fraction can be isolated in budding yeast populations after glucose exhaustion at high density by density gradient centrifugation and cell sorting based on size [25, 28]. Q cells may be a protective or survival strategy, allowing yeast cells to endure unfavorable conditions and serve as a reservoir of cells that can re‐enter the cell cycle and proliferate once conditions become favorable again [26, 29]. Alternatively, the production of Q cells and their lack of division is a non‐adaptive consequence of the inability to reach the appropriate size to enter the first cycle under starvation conditions, rather than a separate program.

### 1.4 Support for Template Strand Co‐Segregation

DNA replication is semi‐conservative [30]. The two strands of DNA unwind during the S‐phase, and each strand serves as a template to synthesize a complementary strand. If a mutation occurs during replication, initially, it will result in a heteroduplex, but, if not repaired, in the next cell division, the mutation will be fixed in one of the daughter cells. The two strands of each chromosome can thus be distinguished from each other based on their relative ages [31]. One strand was synthesized one or two generations or potentially many more generations earlier than the other strand. All chromosomes are therefore made up of complementary strands of varying ages, each of which serves as a template for the subsequent round of DNA synthesis.

**FIGURE 4:**
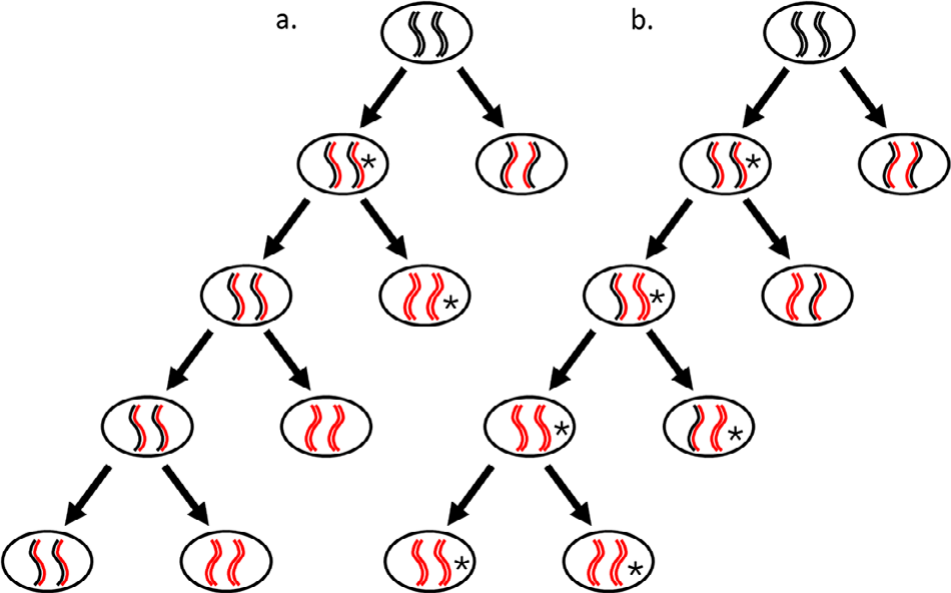
Illustration of template strand segregation and its consequences for the mutation rate during linear growth. (a) A scenario where the template DNA strands (black) are selectively retained by the mother cells that continue to divide. The newly synthesized copy strands (in red), potentially containing mutations due to replication errors, are inherited by the non‐dividing daughter cell. A mutation that occurs during replication (indicated with an *) ends in one cell only, the daughter cell, and not in the mother cell that continues to divide. This results in a reduced number of mutant cells. (b) If there is no selective retention of the template strands, a copy strand can be inherited by a mother cell, in which case a mutation will be inherited by all subsequent daughter cells, thus resulting in a higher number of mutant cells.

John Cairns proposed the “Immortal Strand Hypothesis” (ISH) for mammalian stem cells [32]. According to the ISH, the two daughter cells formed from a dividing stem cell have a divergent cell fate [33]. One of them retains the stem‐cell function and inherits the template‐DNA strands, which are referred to as the “immortal strands” because most mutations are caused by replication errors and hence will be confined to the copy strands (Figure 4a). The other cell undergoing terminal differentiation to form unique mature tissue inherits the newly synthesized copy‐DNA strands, which can have mutations due to replication errors [34, 35]. According to the ISH, all the sister chromatids containing the older strands as the templates are inherited by one daughter cell, while the ones with the younger template strands are inherited by the other daughter cell [36].

The asymmetry in template and copy‐strand DNA inheritance during cell division was initially proposed as a potential mechanism for reducing cancer risk by minimizing somatic mutations in stem cells of animals. The ISH has since been experimentally investigated across diverse cell types and organisms [32]. Quantitative analysis of label retention in mouse embryonic fibroblasts showed that the older template strands labeled with ^3^H‐Thymidine (^3^H‐Td) segregated together [37]. Rosenberg and Kessel noted this distinctive behavior in *Aspergillus nidulans*. They found that nuclei harboring older templates were consistently dispersed to the hyphal tip after mitotic divisions. Importantly, this observation was verified through radioautograms of hyphae treated with ribonuclease in mutants auxotrophic for purines. Moreover, their findings suggest that the system dispersing newly generated nuclei within the hyphal cells is capable of differentiating DNA strands based on their age difference [35]. Over the past six decades, many independent studies have attempted to verify the non‐random DNA segregation in humans and mice [19, 31, 35, 38–40]. Potten et al. conducted labeling experiments with thymidine in the intestinal crypts and tongue papilla. Their results illustrated that specific cells, particularly stem cells at the base of the tongue and in intestinal crypts, exhibited selective retention of older DNA strands [41]. Concurrently, Conboy et al. found that each of the daughter cells had differential cell fates based on the age of the template strand they inherited. The daughter cells inheriting newer strands acquired a differentiated phenotype, whereas those with older templates retained the immature phenotype [40]. Further, independent labelling experiments conducted in human cardiac c‐Kit^+^ stem cells, lung cancer cells, liver, and gastrointestinal cancer cells confirmed the phenomenon of biased segregation of DNA [42, 43, 44]. These results provide substantial evidence supporting the existence of asymmetric template strand inheritance in some tissues.

However, other studies suggested that the segregation of sister chromatids is random, thus contradicting the ISH (Figure 4b). Neff and Burke investigated the mitotic segregation of chromatids following the same basic design of Williamson and Fennel in budding yeast (*S. cerevisiae*) [45]. They labeled the cells with BrdU and measured the intensity of fluorescence in the daughter cells after cell division. In their experiments, they did not observe asymmetric strand inheritance as seen in radioautograms of Williamson and Fennel [45, 46]. A few other studies in mice stem cells also refuted the selective retention of template strands [47, 48, 49]. Furthermore critics contend that asymmetric segregation of chromatids might indeed occur, but for other purposes than for reducing the mutation rate, such as epigenetic marking [50]. Also, sister‐chromatid exchange, the exchange of DNA segments between the sister chromatids during DNA replication, erodes the difference between template and copy strands, thus reducing the efficacy of template‐strand inheritance by stem cells as a mode to reduce the mutation rate. Dalgaard and Klar even argue that sister chromatid exchange protects stem cells from the accumulation of mutations rather than asymmetric template strand inheritance. Stem cells can eliminate harmful mutations by allowing for the exchange of DNA segments, thereby maintaining genomic stability [51]. Dalgaard and Klar also suggested epigenetic marking as another possible mechanism for the reduction of mutation rate. It means that daughter cells inherit epigenetic marks from the parent cell. These epigenetic marks, such as DNA methylation or histone modifications, can influence gene expression patterns and potentially contribute to the differentiation and specialization of daughter cells [50, 51].

So there is some support for the hypothesis that template and copy DNA strands are distributed asymmetrically between stem cells and differentiating cells, but no conclusive evidence [50]. We propose that a mechanism similar to ISH may operate in budding yeast as a protective strategy against mutations due to replication errors during cell division. Replication errors are a significant source of mutations [52] and the proposed mechanism of template‐strand co‐segregation in a dedicated germline can be an effective way of reducing the mutation rate due to replication errors. However, the ease with which asymmetrical inheritance can evolve and the costs it entails, relative to other mechanisms to reduce the mutation rate such as improved proofreading, determine whether the power of natural selection to reduce the mutation rate in this way is sufficient to overcome the effects of genetic drift [53, 54].

### 1.5 Timing of Germline Segregation in Budding Yeast

**FIGURE 5:**
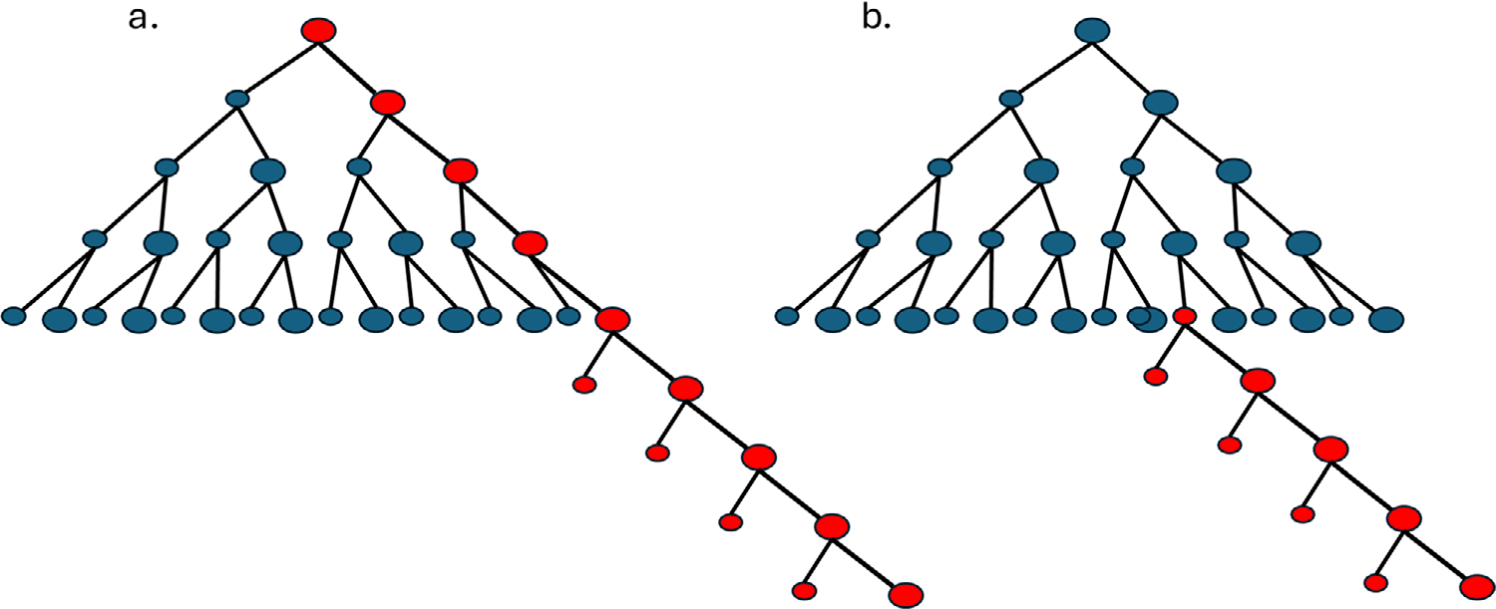
Illustrations of predicted patterns of germline segregation in yeast. The dividing mother cells are indicated with a larger size than budding daughter cells. Cells that are part of the germ‐track are indicated in red, while all other cells are colored blue. (a) A germline is sequestered from the very first division. After the diauxic shift, this cell lineage continues to produce quiescent “offspring” cells. (b) Segregation of the germline occurs from a random cell, after the diauxic shift, when growth shift from exponential to linear.

The timing of germline‐soma differentiation determines the point at which mutations occurring in some cells can no longer be passed on to the next generation (Figure 5). There are three potential relevant moments for the timing of germline segregation in budding yeast: (i) from the first cell division on, a germline lineage remains distinct from the somatic cells formed after every cell division (Figure 5a); (ii) from random cells just after the diauxic shift during the linear phase (Figure 5b); and (iii) intermediate, from a random lineage at a certain point during the exponential growth phase.

The earlier segregation of a yeast germline occurs, the lower the likelihood of mutations being inherited. If early germline segregation occurs, budding yeast cells in particular and unicellular organisms in general may be more similar to multicellular organisms than previously thought. However, it is also possible that the segregation of a yeast germline occurs later, for example, in a random fraction of cells exactly at the diauxic shift. This timing would imply that until this point, all cells potentially can transmit mutations to the next generation, but after the establishment of the germline, only mutations in that designated germline cells are heritable. In the third scenario, the yeast germline could segregate at a random time point during exponential growth. This point could be triggered by environmental factors, cell cycle events, or stochastic processes. Late segregation, whether at the diauxic shift or at a random point, may allow the accumulation of mutations in the population during exponential growth. However, by eventually distinguishing a germline lineage, even at a later stage, the overall mutation rate is reduced compared to a system with no germline segregation at all. Furthermore, the timing of germline segregation might vary both between and within species. It is interesting to consider differences in replicative lifespan between species. For example, fission yeast (*S. pombe*) has a replicative lifespan of only ∼9, which contrasts to 26 of budding yeast [9]. Since such differences have minimal effect on exponential growth (see Supporting Information), it is tempting to speculate on differences between species in the relative importance of germline sequestration during linear growth, for which continued growth of the senescing mother cells is required.

### 1.6 Why Would a Fraction of the Cells Become Somatic?

According to our hypothesis, the somatic cells produced during fermentative growth do not produce any offspring. This fraction of cells thus sacrifices themselves during the fermentative growth phase since they are at an evolutionarily dead end. The formation of heterogenous cell fractions facilitates division of labor among cells [55], but what indirect benefits could the somatic cells provide to the germline cells and can kin selection favor such traits?

One hypothesis is that the main function of cells produced during fermentation is environmental deterioration [24, 56]. Fermentation deteriorates the environment by producing ethanol, a byproduct not generated during aerobic respiration. Besides ethanol production, fermentation also releases heat and CO_2_ faster than aerobic respiration, potentially leading to their accumulation. *S. cerevisiae* may gain a selective advantage if it can withstand such ethanol‐rich, hot, and anoxic environments generated by its fermentation [57]. The somatic fraction of the yeast‐cell population thus could enhance the survival and reproductive success of the clonally related germline cells by reducing competition for resources.

**Glossary Germline Terminology Applied to Budding Yeast**

**Table:** 

Yeast germline	Dividing mother cells after the diauxic shift exclusively committed to producing offspring cells.
Yeast‐germ stem	Yeast cells that can produce both somatic cells and offspring cells.
Yeast‐germ track	The cellular path including germ stem and germline.
Yeast somatic cell	Most cells formed during fermentation, which lose the ability to reproduce after the diauxic shift.
Quiescent yeast cells	Dense cells retaining viability and the ability to reproduce after starvation, characterized by a thickened cell wall, decreased metabolic rate, transcription and translation, and accumulation of a variety of storage molecules [25]. In this paper hypothesized to be unbudded daughter cells formed after glucose exhaustion.
Non‐quiescent yeast cells	Less dense, heterogeneous, replicatively older, asynchronous yeast cells that rapidly lose the ability to reproduce [25]. In this paper hypothesized to be mother cells continuing to divide after glucose exhaustion.
Yeast offspring cell	Quiescent daughter cell formed after the diauxic shift.
Yeast bud	Daughter cell formed as an outgrowth from the dividing mother cell during asexual reproduction in yeast. It is smaller than the mother cell and rejuvenated
Yeast mother cell	Cell that produces a daughter cell by budding. It is larger than the daughter cell and ages with each division.
Yeast daughter cell	The cell produced from a mother cell by budding.
Chronological life span	The length of time that a mother cell can survive in a non‐dividing, quiescence‐like state.
Replicative life span	The number of daughter cells produced by a mother cell prior to senescence. In yeast, the replicative life span is 26.

The effectiveness of environmental deterioration as an altruistic strategy to help clonally related germline cells, however, depends on the relatedness of the cells within the population, which in turn is determined by the number of founding cells. According to Hamilton's rule, altruism can be favored by natural selection if the reduced personal fitness of an individual cell is compensated by the increased fitness of genetically related cells [58, 59]. In populations with high relatedness because of a single or a few unrelated founding cells, the actions of somatic cells are more likely to benefit clonally related germline cells, making this strategy potentially advantageous. In contrast, in populations with low relatedness due to multiple founding cells, altruistic actions will also benefit non‐clonally related germline cells and thus be less likely to be kin‐selected. Surprisingly little is known about the natural ecology of yeast and a key question is the number of founding cells of natural temporary niches of yeast [24].

The second main hypothesis for the function of cells produced during fermentation is the rate‐yield trade‐off [60]. Fermentation allows a faster population growth than respiration since fermentation releases energy faster than aerobic respiration [60]. With competition between unrelated cells, cells with the fastest growth will outcompete the other cells, even if they do not use the resource most efficiently [24]. Fermentation yields only 2 ATP molecules per glucose molecule during glycolysis, whereas respiration can theoretically produce up to 38 ATP molecules per glucose molecule [61]. A proximate mechanism for the rate‐yield trade‐off is protein limitation [62]. Proteins are crucial for both metabolic processes and biomass synthesis, and fermentation is more catalytically efficient in terms of ATP production per protein invested. This efficiency is particularly advantageous under conditions of protein limitation. However, the catalytic efficiency of fermentation comes at the cost of reduced biomass yield [63]. This trade‐off between the rate of ATP production and biomass yield is a critical aspect of yeast metabolism. Fermentation allows for rapid ATP production, which can be beneficial for immediate energy needs, but it limits the overall biomass accumulation compared to respiration [64]. The division of labor among yeast cells by forming distinct soma and germline lineages after the diauxic shift, could potentially help mitigate the effects of protein limitation during respiration. The mother cells continuing to divide during the linear phase can maintain the full complement of protein machinery required for respiration, while their quiescent daughter cells do not need to invest in this machinery. In contrast to environmental deterioration, which we predict depends on high relatedness of yeast cells, theory shows that the rate‐yield hypothesis works under conditions of low relatedness, since competition between unrelated cells favours cells that grow fast but have a lower yield, thus causing a tragedy of the commons [60].

This manuscript focuses on the consequences of asymmetries in DNA‐template strand inheritance during asexual growth of budding yeast and not on the sexual cycle. Under conditions of stress, diploid cells can enter the sexual cycle, undergoing meiosis and producing four haploid spores, which can subsequently mate [65]. Budding yeast is typically diploid in the wild, likely because it frequently encounters nutrient stresses in its natural environment [66, 67]. Despite their wide occurrence, quiescence is understudied in diploid cells, with most research focusing on haploid laboratory strains. To survive prolonged periods of nutrient stress, diploid yeast cells have two strategies. They can either enter a quiescent state or undergo meiosis to produce haploid spores under nitrogen starvation or in the presence of only a non‐fermentable carbon source [68, 69, 70]. Assuming that there is a phase of linear growth in diploid yeast after the diauxic shift, preceding meiosis, the results of this paper can therefore be extended to diploid yeast undergoing meiosis upon starvation.

### 1.7 Testing the Yeast Germline Hypothesis

The yeast germline hypothesis poses some outstanding questions: Does a specific growth phase trigger the formation of a germline? Are the same signals used for germline formation as for the diauxic shift and the onset of linear growth? What role do the somatic cells play, given that they sacrifice their ability to form offspring? Do these somatic cells serve specific functions that are beneficial in natural conditions?

Our hypothesis comprises two major testable components: first, determining whether a distinct fraction of yeast cells forms the germline during the linear phase and if so when this differentiation occurs, and second, examining whether the template DNA strands are selectively retained in the senescing mother cell of the germline lineage. To investigate the first component, microfluidic chips equipped with single‐cell traps or channels can be employed to isolate and observe individual yeast cells [71]. This setup allows for the precise assessment of cell‐division rates, as the number of cells that continue to divide can be closely monitored. Additionally, high‐resolution time‐lapse microscopy combined with automated image analysis software offers a powerful tool for continuously imaging the cell culture over time [72]. This approach enables the tracking of individual cells and their divisions, providing detailed data on the formation of new cells and their lineage. For the second component, the inheritance of the template strands can be studied using DNA methylation marking. This technique involves marking the template DNA strand with methyl groups at specific sites, either by incorporating methylated nucleotides or utilizing site‐specific methyltransferases [73]. Following cell division, methylation patterns of the dividing mother can be analyzed through bisulfite sequencing or the use of methylation‐sensitive restriction enzymes. Bacterial Dam methylase, a well‐characterized DNA methyltransferase produced by *E. coli*, which methylates adenine residues at GATC sites can be used [74]. It has been demonstrated that Dam methylase can function in yeast, enabling researchers to use this system to track DNA replication and segregation [75]. The original template strands, distinguished by these methylation marks, can thus be identified and tracked across generations [73]. Finally, we also postulated that template‐strand co‐segregation will result in a reduction in mutation rate. To investigate this, the mutation rates of yeast cells have to be investigated in the linear phase by single‐cell whole‐genome sequencing of Q and NQ cells, to test if de novo mutations are mostly formed in the Q cells. Since the mutation rate of yeast may be too low to make this approach feasible, a mutator strain may need to be used.

## 2 Conclusion

In this paper, we propose the hypothesis that there is a germline‐soma differentiation in budding yeast, *S. cerevisiae*. Unlike multicellular organisms, which can reduce the number of mutations in germline cells by either establishing a distinct germline early in development in some animals or by reducing the number of cell divisions in cells that form the germ cells in plants, yeast cells may exhibit a unique mechanism during a specific growth phase to protect their genetic integrity. We hypothesize that a small fraction of yeast cells enter a linear growth phase, during which they establish a true germline, devoted to producing quiescent “offspring” cells. These germline cells, which preferentially retain template DNA strands, continue to produce offspring until they are aged, while other cells potentially cease division or take on a somatic role. This growth mode and inheritance pattern would reduce the number of mutations in the progeny of germline cells. Paradoxically, the senescent mother cells thus inherit the mutation‐free template‐DNA strands, while the rejuvenated daughter cells inherit the copy‐DNA strands.

Our hypothesis challenges the traditional view that unicellular organisms cannot segregate a germline from a soma and suggests that yeast cells might employ a form of DNA‐level germline sequestration with some similarities to ciliates. We presented some circumstantial evidence supporting this hypothesis, but the two main components, that is, details of yeast growth mode and asymmetries in DNA‐template strand inheritance during linear growth, remain to be experimentally established, and subsequently, molecular and cellular mechanisms underlying germline segregation can be studied. Furthermore, basic knowledge of the natural ecology of yeast remains to be gained to fully appreciate any adaptive significance of such germline segregation.

## Author Contributions

D.K.A. developed the idea for this manuscript and discussed it with B.S.V.; D.K.A. and B.S.V. both edited and wrote parts of the manuscript.

## Conflicts of Interest

The authors declare no conflicts of interest.

## Supporting information



Supporting Information

## Data Availability

Data sharing is not applicable to this article as no new data were created or analyzed in this study.
