# Supplementary material for: Can Unicellular Organisms Sequester a Germline? The Yeast‐Germline Hypothesis
Source: Bioessays. 2025 May 2;47(6):e70003. doi: 10.1002/bies.70003 (PMC12101049; doi:10.1002/bies.70003)
Supplement: Supplementary file 1 — Supporting Information [file BIES-47-e70003-s001.docx]

**Supporting Information**

Assuming optimal culture conditions, a single budding-yeast cell entering exponential growth can divide 26 times, corresponding to 67.1 million cells. The effect of cell-level senescence is negligible, since at that point in time, the first cell of the 67.1 million cells produced will then die due to senescence. To understand the consequences of cell-level senescence on population growth for cases with faster senescence, we simulated growth curves for unicellular organisms with fewer cell divisions. One extreme is that a cell can undergo only one cell division, in which case the number of cells remains constant. With each additional potential division before senescence starts, the population size increases, but less than the maximum possible with infinite growth without senescence. Our simulations illustrated in **Fig 1** show that cell death in a population with more than six possible cell divisions has minimal effect on overall population growth compared to a scenario where there is no cell-level senescence. This shows that the effect of cell-level senescence on population growth is minimal from more than six cell divisions onwards.


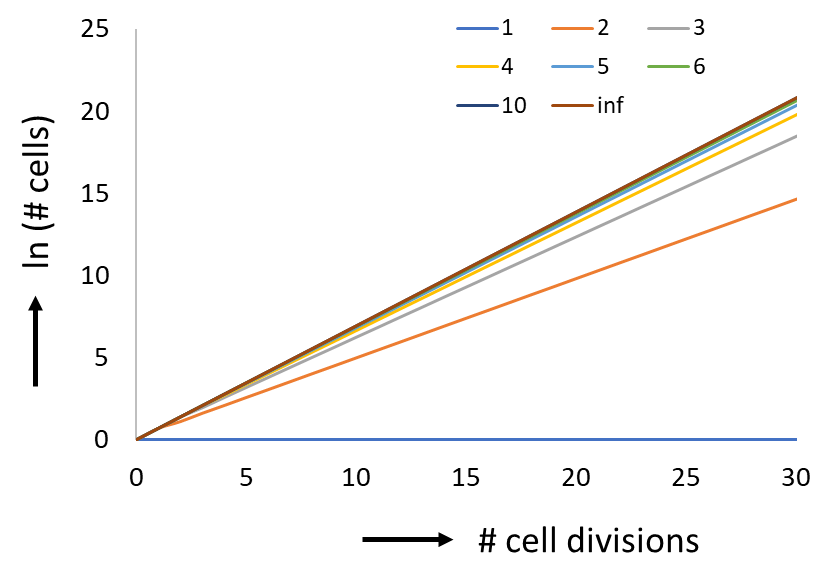


Figure 1. **Simulations of growth curves of budding yeast populations** when the cells divide only once (dark green curve), twice (blue curve), 3 times (purple curve), 4 times (light green curve), 5 times (dark blue curve), 6 times (brown curve), 10 times (black curve), no senescence (orange curve).
